# Fibrin/MoS_2_-nanosheet conductive hydrogels with programmed time scales and pathways for bioresorption

**DOI:** 10.1039/d5na00377f

**Published:** 2025-08-21

**Authors:** Vidushi Shukla, Willis T. Bilderback, Deisy Fernandes, Mark Daley, Rojry Basnet, Pushkaraj Joshi, Zidan Yang, Anubhav Tripathi, Jacob K. Rosenstein, Kareen Coulombe, Robert H. Hurt

**Affiliations:** a School of Engineering, Brown University Providence Rhode Island USA Robert_Hurt@brown.edu; b Department of Chemistry, Brown University Providence Rhode Island USA; c Institute for Biology, Engineering, and Medicine (I-BEAM), Brown University Providence Rhode Island USA

## Abstract

Electrically conductive hydrogels are of interest as scaffolds for tissue engineering applications involving the growth, implantation, or attachment of electrically active cells. Such hydrogels should exhibit soft mechanics, tunable conductivity to match native tissue, biocompatibility, and biodegradability into non-toxic, clearable species. Common conductors based on metals or polymers can be challenged by insufficient biocompatibility or biodegradability. A potential new alternative is the use of composites containing 1T-phase MoS_2_ nanosheet fillers, which have a metallic nature and undergo oxidative biodegradation over clinically useful time scales. Chemically exfoliated MoS_2_ is introduced into assembly protocols for fibrin hydrogels and the composites characterized by electrochemical impedance spectroscopy, which reveals a 400% increase in conductivity in the physiologically important mid-band region of 10^3^–10^4^ hertz. *In vitro* studies on fibrin/MoS_2_ composite hydrogels show complex multipath biodegradation behaviors. Matrix metalloprotease action degrades fibrin to soluble protein, without attacking the nanosheets. The nanosheets degrade separately by H_2_O_2_ oxidation to soluble molybdate in a self-limiting reaction inhibited by the catalysis of peroxide decomposition by the molybdate product. Genipin cross-linking is demonstrated as a method to stabilize the fibrin network, control the overall hydrogel monolith lifetime, and control the biodegradation pathway to avoid nanosheet release by early loss of the fibrin network. The composite degradation products were found to be non-cytotoxic to primary cardiac fibroblasts by the MTT assay. Overall, 1T-phase MoS_2_ nanosheets offer an attractive alternative to currently available inorganic or polymeric additives for creating conductive, bioresorbable, and biocompatible hydrogels.

## Abstract 2

MoS_2_ nanosheets in metallic 1T-phase enhance the electrical conductivity of fibrin hydrogels and show programmable bioresorption rates and pathways.

## Graphical abstract

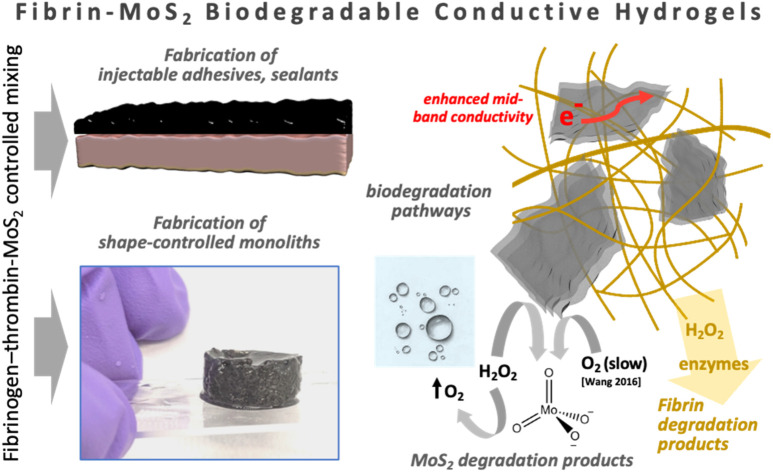

## 1. Introduction

Hydrogels are multiphase, high-water-content, shape-retaining materials, whose soft mechanics and aqueous chemistry allow them to interface effectively to living tissue. Hydrogels are used in established commercial technologies as contact lenses and wound dressings,^1–3^ skin-electrode interfaces for electrocardiograms,^4^ and surgical sealants.^1–3^ They are also under development for myriad biomedical technologies involving drug delivery,^5^ tissue engineering^6,7^ and device coatings that enhance biocompatibility or function.^1^ Hydrogels are either injectable (administered as liquids that gel *in situ*) or non-injectable (pre-assembled gels). Non-medical applications include water retention agents in agriculture, environmental sorbents for water-borne contaminants, and delivery vehicles for agrochemicals,^1^ as well as supercapacitors,^8,9^ shape morphing stimuli-response materials,^10^ and microbial scaffolds or containment devices.^1,11,12^

An emerging subclass of hydrogels are those designed for enhanced or tunable electrical conductivity (see review by Kougkolos *et al.*^13^). Conductive hydrogels find use in sensors and soft actuators,^1,10,11,14^ ionic conductors,^7^ and technologies for repair of electroactive neural, cardiac, or muscle tissue.^13,15–19^ The use of conductive scaffolds with or without electrical stimuli in tissue engineering have been shown to affect cellular activities including cell adhesion, migration and proliferation.^18,19^ Scaffolds with electrical conductivity near the conductivity of the native tissue are often most effective.^13,21^ Conducting hydrogels typically use hydrophilic polymers as primary network formers, which are doped with conducting polymers such as polythiophene, polyaniline, or polypyrrole.^20,22^ Challenges for the use of conductive polymers in tissue regeneration include avoiding adverse cytotoxic responses and the need for chemical modification to impart biodegradability.^18,20,23^ In one approach, Guo *et al.*^20^ engineer biodegradability by introducing degradable segments to conductive polymers, while and other approaches limit cell exposure by integrating the conductive polymers into other polymer matrices.^18,24,25^

Alternatives to conducting polymers include inorganic filler materials such as gold nanorods, graphene nanosheets and carbon nanotubes.^12,26,27^ While inorganic filler materials can enhance the electrical and mechanical properties of hydrogels, many are persistent rather than bioresorbable and/or raise biocompatibility concerns for *in vivo* applications.^18,28,29^ An ideal conductive filler for implantable tissue engineering applications would possess (i) intrinsically high electrical conductivity, (ii) a high aspect ratio (for efficient solid network formation), (iii) low toxicity in the solid state, (iv) hydrophilicity (useful in hydrogel synthesis), and (v) time-scale-tunable biodegradability into degradation products that have low toxicity and are excretable or resorbable. Few candidate filler materials offer this complete property set.

Here we explore a new class of resorbable conductive hydrogel based on metallic-phase transition metal dichalcogenide (TMD) nanosheets. One member in this class is the 1T metallic phase of 2D molybdenum disulfide, MoS_2_ which has been reported to biodegrade by enzymatic action,^30^ or by non-catalytic O_2_ oxidation,^31,32^ which relies only on this ubiquitous oxidant in biological and environmental settings. The literature suggests it meets all five of the above criteria:

(i) Intrinsic electrical conductivity. Though the most common and stable phase of MoS_2_ is the trigonal prismatic 2H semiconducting phase, chemical exfoliation methods used to isolate the atomically-thin nanosheets convert a significant fraction of the material to the octahedral 1T phase, in which atomic rearrangements within the nanosheet gives rise to metallic behavior with electrical conductivities 10^7^ times higher than the semiconducting 2H phase.^33–36^ Hydrothermal and solvothermal processes have also been used to make MoS_2_ in metallic phase.^35^ The exfoliation protocol used here yields a mixed phase of about 60% metallic 1T phase and 40% semiconducting 2H phase determined by XPS.^31^

(ii) High aspect ratio. Monolayer MoS_2_ nanosheets have three atomic planes (S–Mo–S) and a thickness of ∼1.2 nm. Chemical exfoliation methods are reported to yield high concentrations of these monolayers with lateral dimensions on the order of 250 nm or greater^31,36^ corresponding to aspect ratios on the order of 200 or greater.

(iii) Low toxicity in the solid state. Among inorganic materials, MoS_2_ is regarded to pose a relatively low human health hazard.^37–39^ Direct toxicity assessment on samples free from contamination suggests that MoS_2_ nanosheets have a low cytotoxicity that make them suitable for applications in flexible biosensing/bioimaging devices and biocompatible coatings.^37^

(iv) Hydrophilicity. 1T MoS_2_ nanosheets are hydrophilic and water dispersible^31^ with a negative surface charge greater than −45 mV zeta potential at pH values greater than 2.0.

(v) Biodegradability. The use of nanomaterials in clinical applications require careful consideration of biocompatibility, biodegradation, and ultimately biodistribution.^40^ Many inorganic materials when exfoliated into atomically-thin 2D forms show high rates of biodegradation (see recent reviews^32,41^). Bulk MoS_2_ forms surface oxides during atmospheric air exposure, while 1T-phase MoS_2_ nanosheets in air-saturated aqueous media undergo complete oxidation to soluble products.^31^ The oxidation rate is dependent on pH, crystal phase (1T *vs.* 2H), and the presence of reactive oxygen species (ROS), and occurs over time scales from days to months.^31,32^ Oxidative degradation of MoS_2_ produces molybdates, which are low-toxicity soluble ionic species.^32^ Molybdenum is an essential trace element, and the metabolism of 2D MoS_2_ nanodots has been reported to deliver bioavailable Mo to molybdenum enzymes *in vivo*.^42^ The air-sensitivity of MoS_2_ is an obstacle for electronic device applications, but is a potential advantage in tissue engineering if degradation rates and solid-state lifetimes can be controlled.

While MoS_2_-based 2D materials have been used in a number of biomedical studies,^38,43–51^ as well as energy/environmental studies,^9,52,53^ the use of the metastable metallic 1T phase as a conductive bioresorbable filler has not been previously reported.

The present work fabricates self-assembled fibrin hydrogels doped with MoS_2_ nanosheets obtained from lithium-insertion chemical exfoliation with majority 1T phase. Fibrin nanofiber matrices are natural tissue constructs that form during wound healing by reaction between fibrinogen, a 340 kDa glycoprotein that is the circulating inactive precursor of fibrin, and the serine protease thrombin, which is activated in response to injury.^54^ Thrombin cleaves the fibrinopeptides to convert soluble fibrinogen to fibrin monomers that self-polymerize into longer oligomers and then aggregate and branch to create a 3D fiber network. *In vivo* the fibrin network is further stabilized by Factor XIIIa, which covalently binds glutamine residues in one fibrin molecule to lysine residues in another, making the gel or clot more stable against mechanical forces and chemical degradation. Fibrin exhibits both biocompatibility and biodegradability through enzymatic degradation and resorption; and it has bio-adhesive properties. In biomedicine, fibrin-based hydrogels already find application as surgical glues, wound sealants, and scaffolds for tissue engineering.^55–57^ Here we explore composite fibrin hydrogels with MoS_2_ nanosheets as a conducting filler phase, and characterize their frequency-dependent electrical conduction and their kinetics and pathways of biodegradation, including control of reaction timescales and intermediates.

## 2. Results and discussion

### 2.1 Hydrogel fabrication and morphology

**Fig. 1:**
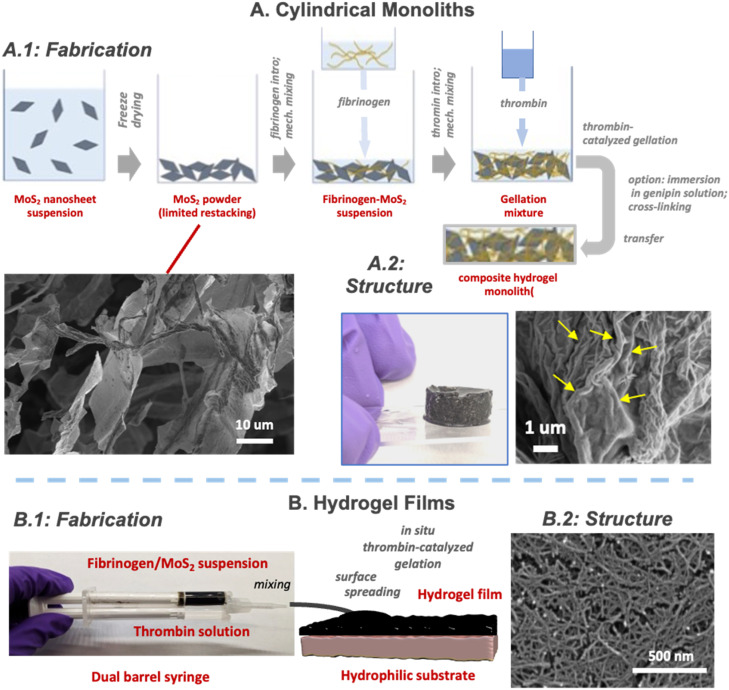
Fabrication approaches and structures of fibrin/MoS_2_-nanosheet composite hydrogel materials. (A) Cylindrical monoliths. (A.1) Fabrication steps to prepare cylindrical monoliths, and an SEM image of the intermediate pure MoS_2_ dry powder after freeze drying. (A.2) Structures of the composite hydrogel monoliths revealed by optical imaging, and by SEM following sample preparation by critical point drying. The yellow arrows show a nanofibrous structure, which is characteristic of the fibrin component. (B) Hydrogel films. (B.1) Film fabrication method adapted from the surgical application of fibrin glues and based on use of a double barrel mixing syringe. (B.2) Top view of hydrogel films by critical-point-drying/SEM again showing self-assembled fibrin nanofibers.

The electrical characterization and biodegradation studies in this paper were performed on cylindrical gel monoliths, whose fabrication methods and morphology are illustrated in Fig. 1. Chemical exfoliation was used to prepare majority 1T-phase MoS_2_ nanosheets by a published protocol (see Methods). These nanosheets are observed to be primarily monolayers with lateral dimensions from 200–500 nm (Fig. S1) and XPS analysis (Fig. S2) indicates a mixture of 1T (majority) and 2H (minority) phases, consistent with previous published results.^31^ Dilute stock suspensions of these nanosheets were first concentrated by freeze-drying to produce a pure MoS_2_ powder (Fig. 1A.1). Thermal drying was attempted first but caused massive restacking to a single dense metallic grey MoS_2_ film that was difficult to redisperse. Freeze drying did not fully eliminate re-stacking, but limited its effects to local aggregation of nanosheets into micron-scale flakes, which exist in a randomly aligned state in a macroscopic, fluffy, low-density powder (Fig. 1A.1). Hydrogels were prepared by filling a glass mold in sequence with freeze-dried MoS_2_ nanosheet powder, fibrinogen solution, and then thrombin solution under stirring. Gellation was observed to occur within one minute. Pure fibrin monoliths are clear, while those with 12 dry-wt% MoS_2_ were uniformly black and opaque (Fig. 1A.2). Critical point drying followed by SEM imaging reveals a nanofibrous structure (yellow arrows, Fig. 1A.2), which is characteristic of the fibrin component.^45,47^ The gels (∼2 cm diameter × 1 cm height cylindrical pucks) remained in the mold for electrical testing (see below), with electrical leads introduced on the top and bottom, while for biodegradation studies, the gels were detached and physically removed to allow all-sides access by the degradation media.

Films were also explored as an alternative gel form (Fig. 1B) of interest in applications involving device coatings, sealants or adhesive functions.^1–3,55,56^ The fabrication method was adapted from fibrin surgical glue protocols,^55,56^ in which a double-barreled syringe with a mixing tip is used co-deliver fibrinogen and thrombin solutions to a substrate (Fig. 1B.1). Mixing at the syringe tip homogenizes the liquid and contacts fibrinogen with thrombin to initiate polymerization leading to precipitation of insoluble fibrin and gelation into a gel film^55,56^ (Fig. 1B.1 and S3). The substrates were 1′′ × 3′′ glass slides or PET sheets treated with O_2_-plasma for hydrophilicity to promote liquid spreading. Various approaches were explored to introduce the MoS_2_ nanosheet component into the established fibrin glue procedure, and a simple T-peel adhesion test^58,59^ was used to provide feedback on the quality of the assembled fibrin network (Fig. S4 and associated SI text).

Premixing MoS_2_ and thrombin solutions led to problems with colloidal destabilization and aggregation of the nanosheets. We reported previously,^31,60^ that 1T phase MoS_2_ nanosheets prepared by this chemical exfoliation method are colloidally stabilized by electrostatic repulsion through negative electric surface charge (zeta potential −50 mV in pH 7.5 aqueous suspensions). Here the thrombin solutions are formulated in phosphate buffered saline (PBS) with 0.9 wt% NaCl that we observed to destabilize the colloid by the well-known effect of surface charge screening. We attempted to reduce the ionic strength by substituting PBS, but thrombin is a Na^+^ activated enzyme,^61^ and reducing Na^+^ led to inhibition of gelation and low strength in adhesive strength testing (Fig. S4). Introducing MoS_2_ to the fibrinogen barrel (Fig. 1C) was more successful (Fig. S4), and interestingly the MoS_2_/fibrinogen colloidal suspension had fewer aggregates even in the presence of NaCl. Fibrinogen has been reported to form protein coronas on nanomaterial surfaces in aqueous media^62,63^ and to provide colloidal stability through that corona formation,^63^ which is the likely effect we observe. Fig. 1B.2 shows an SEM image of the film top surface after critical point drying, showing a nanofibrous morphology, as expected.

### 2.2 Electrical property characterization

First the electrical conductivity of pure MoS_2_ nanosheet films was characterized by applying a four-point probe method. The films were formed by solution casting of 1 mg per mL MoS_2_ stock suspensions onto quartz slides followed by 60 °C oven drying. The films were metallic grey, smooth, possessed no observable macro-porosity, and had lateral dimensions of ∼2 × 3 cm and thicknesses of about 3.6 μm, corresponding to ∼10^3^ MoS_2_ layers. A four-point probe with adjacent probe spacing of 2.5 mm gave an electrical resistance of 0.25 kOhms, which corresponds to a sheet resistance of 1.0 kOhms per square using the standard 4-pt probe model equations summarized by Miccoli *et al.*^64^ with corrections for finite thickness and lateral dimension. The intrinsic conductivity of this pure MoS_2_ nanosheet film was calculated from the thickness as 272 S m^−1^. This value is similar to other as-produced MoS_2_ films made by the Li intercalation method.^34^ For example, Acerce *et al.*^36^ cite 1T MoS_2_ conductivities in the range of 10^3^–10^4^ S m^−1^, while the present mixed-phase 1T/2H material has an overall conductivity just below this range, as might be expected.

Pure fibrin hydrogels fabricated using salt buffers are expected to be ionic conductors with resistivity influenced by the dissolved ion concentrations and mobilities. Addition of MoS_2_ introduces electron-conducting nanosheets, which have high local conductivity, but do not likely form a contiguous percolating electron-conducting path at the MoS_2_ volume fractions used here (10–15 dry-wt%). Even without complete electron conduction paths, the nanosheets and aqueous ions are capacitively coupled, and electrical impedance spectroscopy (EIS) becomes a useful tool to characterize the frequency-dependent electronic properties.

**Fig. 2:**
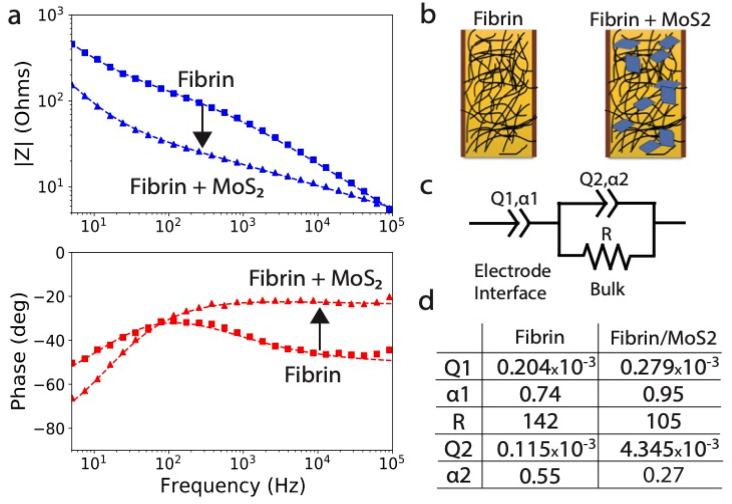
(a) Measured electrochemical impedance spectra (EIS) of the fibrin-based hydrogel and fibrin/MoS_2_ composite hydrogel as a magnitude and phase Bode plot; (b) a pictorial representation of fibrin hydrogel structure with and without MoS_2_ nanosheets; (c) electrical circuit equivalent used to model the EIS spectra; (d) fitted parameters of the equivalent circuit model with complex impedance shown in eqn (1).

Fig. 2 shows EIS measurements of the pure fibrin and fibrin/MoS_2_ composite gels in the cylindrical monolith format. In the physiologically important mid-band range between 100–1000 Hz, the MoS_2_ addition increases conduction by about 400%, from approximately 0.3 mS cm^−1^ to 1.3 mS cm^−1^. At higher frequencies, the MoS_2_ addition also reduced the phase shift from −50° to −25°. The corresponding Nyquist plot is shown in Fig. S5. The EIS data can be modeled with an equivalent circuit containing a constant phase element (CPE) representing the electrode interface (Q1), and a parallel CPE and resistor representing the bulk hydrogel (Fig. 2c). Fig. 2d shows fitted parameters of the equivalent circuit model with complex impedance:1
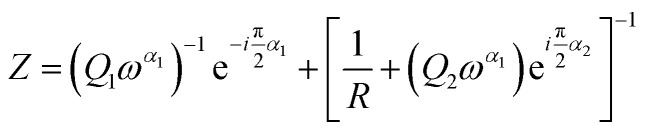


The decreased mid-band impedance and reduced phase shift are consistent with improved electrical conductance due to contributions from the high-electron-conductivity MoS_2_ filler phase. These results compare favorably to the enhancements achieved by other conductive additives.^13^ For example: Liu *et al.*^65^ used reduced graphene oxide and carbon nanotubes to increase the electrical conductivity of oligo(polyethylene glycol) fumarate hydrogels from 0.002 mS cm^−1^ to 0.0079 mS cm^−1^. Xia *et al.*^27^ fabricated a conductive polyacrylamide/chitosan hydrogel with carboxylated multi-wall CNTs that increased conductivity from 1.0 to 9.5 mS cm^−1^. MoS_2_ in the 1T phase offers similar conduction enhancement but in a fully biodegradable form with, non-cytotoxic degradation products (see next section).

### 2.3 Biodegradation studies

#### 2.3.1 Kinetics and pathways

Degradation of scaffold and matrix proteins in the body can occur by physical (dissolution), chemical (hydrolysis) and/or biological processes (enzymatic or immune response related).^66^ Typically, enzymatic activity is the largest driver of biodegradation, with serine proteases (*e.g.* trypsin, plasmin) and matrix metalloproteinases (*e.g.* collagenases) seen as the most likely enzyme classes.^67^ The rate of degradation *in vitro* greatly affects the utility of bio-scaffolds – too rapid may leave supported materials vulnerable to immune attack or affect the required activity of a biomedical device, while too slow may cause inflammation or scarring.^66^ Typical target time scales for biodegradation range from days to months, depending on the application.^1^

Nanocomposite scaffolds pose additional complexity. In addition to controlling overall time scale, one is concerned with the relative degradation rates of the two components, which determines the intermediate structures of the scaffold as it evolves over time, and the release or retention of the nanoscale components and their degradation products. In the case of fibrin/MoS_2_ hydrogels, the literature suggests that preferential degradation of the MoS_2_ would release molybdate ions,^31,32^ while leaving the fibrin network and overall hydrogel form intact. In contrast, preferential degradation of the fibrin network could release free MoS_2_ nanosheets into surrounding physiological fluids. While MoS_2_ is a relatively low-toxicity nanomaterial,^30,39^ the potential for unintended nanosheet release would still raise the issue of possible nano-specific biological responses, with their complex dependence on shape, size, and surface properties of the nanoscale solids.

Here we use the cylindrical gel monoliths (Fig. 1A) to study the time scales and pathways for fibrin/MoS_2_ biodegradation. We perform time-resolved *in vitro* studies using a model enzyme for fibrin degradation and a reactive oxygen species for MoS_2_ oxidative degradation, with the active degradation reagents applied both separately and in combination. The MoS_2_ 1T phase is known to undergo O_2_-mediated oxidative dissolution in air-saturated aqueous media^52^ and H_2_O_2_-mediated dissolution in physiologically relevant concentrations of peroxide.^30^ Here we use millimolar concentrations of H_2_O_2_ in air-exposed buffers to model the oxidative pathway, and a collagenase mixture as a model matrix metalloproteinase (MMP) for the enzymatic pathway. The MMPs are a large family of proteolytic enzymes that degrade major components of the extracellular matrix including collagen, fibrin, basement proteins and other glycoproteins.^68^ While the enzyme plasmin is most closely associated with fibrinolysis *in vivo*, we chose a general MMP mixture (bacterial collagenase 1A from *Clostridium histolyticum*) as a convenient model to handle the various modified and composite formulations we envision for these gels. Collagenase degradation of fibrin gels have been reported previously in the literature,^69,70^ but their interactions with MoS_2_ or its degradation products are unknown.

**Fig. 3:**
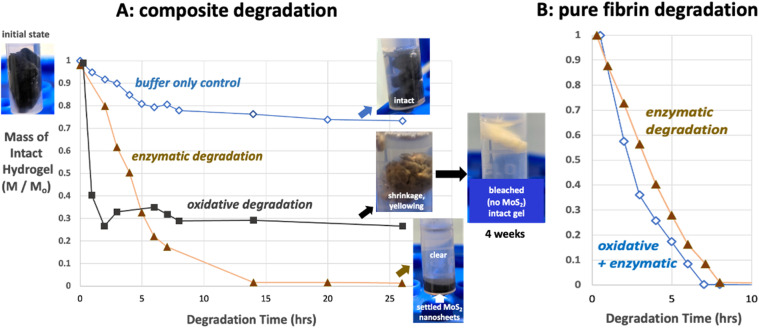
Hydrogel degradation rates and structures for fibrin/MoS_2_ composites (A) and pure fibrin gels (B) along different chemical degradation pathways. Gel monoliths were shaken at 60 rpm in the degradation solutions and the intact portion(s) recovered intact and transferred in the fully wet state to a laboratory milligram balance for mass measurements. Enzymatic degradation was modeled using collagenase (0.5 mg mL^−1^) and oxidative degradation using hydrogen peroxide (0.4 mM), both in TES-Ca buffer.

Fig. 3 illustrates the observed degradation behavior for the composites and for fibrin-only gels as a comparison. In pure PBS some spontaneous mass loss is observed, primarily over the first 5 h, before slowing. In the present of the enzyme alone the gel monolith is destroyed and free nanosheets are liberated, which settle to a dark deposit at the bottom of the vial (Fig. 3). The oxidative pathway was expected to preferentially attack the MoS_2_ nanosheets, leaving intact clear fibrin gels without the dark coloration of MoS_2_. At long times (4 weeks) we do indeed observe colorless gels with their macrostructure intact (Fig. 3A inset), where the bleaching indicates degradation and dissolution of MoS_2_ nanosheets. At earlier times (25 h), however, the gels first turn yellow, not clear, and the nature of this intermediate state was at first unknown. Also unexpected is the large oxidation mass loss in the first two hours, which represents significantly more mass than the 12 wt% original MoS_2_ nanosheet loading. This suggests some mechanism for H_2_O_2_-mediated fibrin degradation in addition to H_2_O_2_-driven MoS_2_ oxidation. The ability of peroxide to degrade fibrin was not anticipated based on current literature. At longer times the oxidative pathway settles at ∼30% mass remaining, which appears to be an asymptotic value. This may reflect depletion of the H_2_O_2_, or some pathway for H_2_O_2_ decomposition by disproportionation. The sections below seek to explain these various data features by identifying the degradation products and exploring the reaction pathways and mechanisms in detail.

#### 2.3.2 Degradation products and reaction mechanisms

Identifying biodegradation products is an important task in the development of safe resorbable biomaterials.^32,71^ We began by studying the degradation products from the two composite components, fibrin and MoS_2_, separately. For MoS_2_ nanosheets, oxidative dissolution processes have been well studied,^30–32^ and includes reports of time scales, reaction kinetics, enzymatic catalysis, and pH dependence,^30–32^ as well as indirect evidence for molybdate ions as the primary product.^31^

For the fibrin component, Fig. 3 suggests that enzymatic, oxidative, and physical pathways all play a role. Fibrin degradation has been extensively studied and in the human body is typically ascribed to enzymatic pathways with plasmin as the most important fibrinolytic enzyme,^54,72,73^ which enables localized, controlled surface degradation of clots. Blood-based assays for fibrin degradation products (FDPs) are used clinically to detect FDPs as biomarkers of conditions that include deep vein thrombosis, pulmonary embolism, and disseminated intravascular coagulation. The slow, non-enzymatic processes (buffer-only control) is similar to the observations of Egorikhina *et al.*^66^ on hybrid fibrin–collagen scaffolds. That study described “passive” degradation in PBS buffers without added reagents, in a process that liberates free protein into solution but at a lower rate than enzymatic degradation.^66^

We could not find literature reports of fibrin degradation by the oxidative route implied in Fig. 3. There are reports that peroxide in the presence of horseradish peroxidase can increase fibrin cross linking,^74^ which may alter fibrin structure. It was not at first clear from the Fig. 3 data alone if the loss of wet mass represents peroxide cross linking and physical contraction (to expel water from the hydrogel), or true chemical degradation to yield dissolved molecular products.

**Fig. 4:**
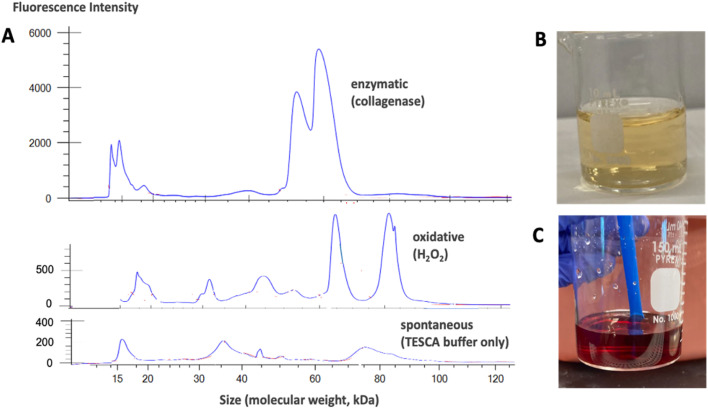
Characterization of the soluble products from biodegradation of fibrin gels and MoS_2_-nanosheets in this study. (A) Molecular weight distribution of soluble protein from the degradation of fibrin gels in three fluid environments: Top: 0.5 mg per mL collagenase in TESCA buffer; 120 mM H_2_O_2_ in TESCA buffer; TESCA buffer alone. (B) Evidence for molybdate as the oxidation product of MoS_2_ nanosheets. A yellow diperoxomolybdate complex^77^ forms after mixing H_2_O_2_ and Na_2_MoO_4_, which reproduces the color seen in the fibrin/MoS_2_ gels and solutions after partial oxidative degradation (see Fig. 6). (C) Higher H_2_O_2_ concentrations yield red solutions consistent with triperoxomolybdate.^77^

We first used filtration to see if the H_2_O_2_-mediated degradation products were cross-linked contracted solid gel fragments or dissolved molecular species. Incubation of pure fibrin gels for 72 h in 120 mM peroxide only (TESCA buffer) caused complete disappearance of the solid gel with no products collected on a 0.45 μm filter, suggesting true chemical degradation to dissolved species. This behavior is not an artifact of the high peroxide concentration used, as we also observe it at 7 mM peroxide under the same conditions. We then used a microfluidic electrophoresis assay^75^ to look for soluble proteinaceous material in the degradation solutions exposed to pure fibrin gels. Fig. 4A shows molecular weight distributions of free (dissolved) protein present after 15 h incubation in the enzymatic, oxidative, and buffer-only solutions. Free protein of MW from 15 to 120 kDa were detected using a protein express ladder to calibrate MW with microfluidic elution time. Based on literature^72^ we would also expect higher-MW products as well, outside of the current range of measurement. The fluorescence intensities varied greatly with pathway (enzymatic > oxidative > buffer-only) and the peaks in the MW distributions also varied, as might be expected from the different chemical pathways involved. Hiller *et al.*^76^ report a variety of soluble products also in the 20–70 kDa range produced by matrix metalloproteinase-mediated degradation of fibrinogen. We note that the oxidative degradation clearly involves free protein release and is not just a physical contraction associated with oxygen cross-linking. It is not clear why peroxide-mediated fibrin degradation has not been reported previously, but we note that this portion of our study used as-produced fibrin without cross linking by Factor XIIIa or other agents such as genipin (*vide infra*), and may thus be more susceptible to oxidative attack than cross-linked fibrin. It is possible that H_2_O_2_-induced oxidation of unstabilized fibrin (without Factor XIIIa or genipin crosslinking) adds polar functional groups to fibrin and reduces the hydrophobic forces between segments that contribute to network cohesion. Fig. 4 also provides information to support a previous hypothesis that claimed soluble molybdates are the main oxidative degradation product of MoS_2_ nanosheets.^31^Fig. 4B shows that fibrin–MoS_2_ gels and solutions acquire a yellow color after partial oxidative degradation, but not the gels following other degradation pathways. To explore its molecular origin, we did combinatorial mixing of our expected degradation products and buffer components and examined the solutions. Mixing sodium molybdate with TES sodium salt, sodium sulfate, sodium chloride, and sodium phosphate dibasic only produced clear solutions. Upon addition of hydrogen peroxide, the solution immediately took on a dark, reddish-brown color, which faded to orange-red after 10 min and to a vivid yellow after 24 h. Adding 40 μL of 30% hydrogen peroxide to five mL of deionized water with 13.4 mg sodium molybdate (to produce concentrations similar to those expected in nanosheet degradation experiments) gave rise to a dull yellow color similar to that observed in the degradation solutions. This result suggests the formation of a complex between molybdate anions and hydrogen peroxide that partitions between the gel and surrounding solution. The literature reports a dominant red-colored triperoxomolybdate species at high H_2_O_2_ concentrations and a dominant yellow-colored diperoxomolybdate species at lower peroxide concentration.^77^ The color change from red to yellow over time occurs in response to the gradual decomposition of H_2_O_2_, which we show later is catalyzed by molybdate (*vide infra*).

**Fig. 5:**
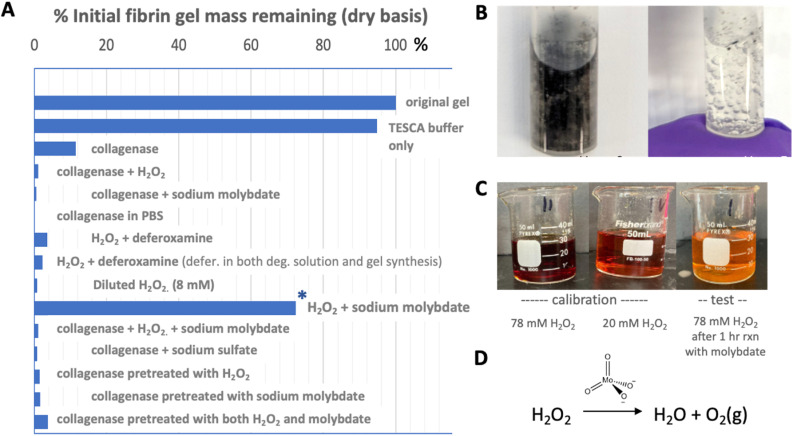
Multispecies interactions affecting degradation of the fibrin gel matrix. (A) Results of auxiliary experiments measuring the extent of degradation mass loss after 3 day incubation in TESCA buffer (pH 7.4) with different combinations of solutes in the fluid medium. (B and C) Evidence for molybdate catalysis of peroxide decomposition in the form of gas evolution and gradual loss of color associated with peroxomolybdate complexes; (D) catalytic disproportionation.

We sought to understand more about the fibrin/enzyme/peroxide/molybdate interactions, by systematically studying them in pairwise combinations. Fig. 5 shows the results of a series of auxiliary experiments using pure fibrin gels, where % degradation was measured on the dry gel mass following incubation in degradation solutions for a standard time period of 3 days. Peroxide alone at 120 mM is capable of complete fibrin degradation. Further collagenase activity is not inhibited by interactions between molybdate or H_2_O_2_, occurring either during the reaction or by pretreatment. Collagenase activity is also not inhibited by change in buffer (from TESCA to PBS). The only component combination that inhibited fibrin degradation was peroxide in the presence of sodium molybdate, and other sodium salts did not produce the effect, so there must be an H_2_O_2_–molybdate interaction. We conclude that H_2_O_2_ attack on the fibrin/MoS_2_ composite gels produces molybdate anions from the nanosheets, which, after sufficient accumulation catalytically destroy the remaining H_2_O_2_. This is confirmed by gas evolution in this system (Fig. 5b), presumed to be O_2_, and easily seen at high peroxide concentration, as well as color loss over time associated with the loss of the peroxide–molybdate complexes (Fig. 5C). Fig. S6 (SI) gives a proposal for the catalytic mechanism. Fig. 5 also shows that the loss of peroxide activity is not due to catalysis by trace iron content since it cannot be restored by addition of the Fe chelator deferoxamine.

#### 2.3.3 Control of degradation time scales and pathways

Depending on the application, target time scales for hydrogel biodegradation range from days to months.^1^ The times observed in Fig. 3 are at the low end of this scale, so we sought an approach to increase hydrogel lifetime. Ideally one would identify a practical modification to the gel synthesis that would increase the resistance of the fibrin component, in particular, and for both the enzymatic and oxidative pathways. This would lengthen the lifetime of the gel macrostructure and ensure that the MoS_2_ nanosheets could complete their oxidative dissolve before the fibrin framework collapses with the potential for free nanosheet release (see pathway “A” in Fig. 8).

**Fig. 6:**
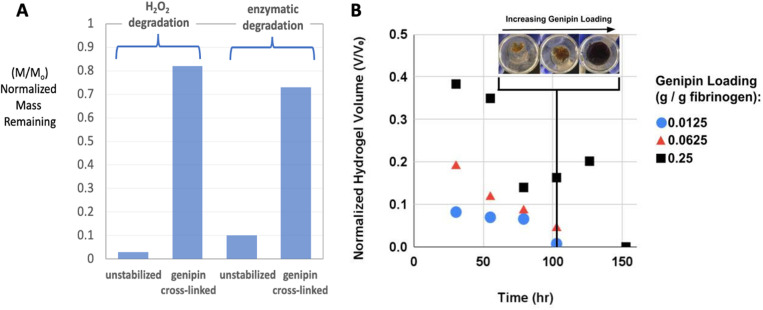
Control of degradation times by fibrin cross-linking. (A) Effect of genipin cross-linking (1 : 4 genipin : fibrin mass ratio) on degradation behavior (mass remaining, dry basis) of pure fibrin gels exposed for 3 days to 120 mM peroxide or 0.5 mg per mL collagenase. The peroxide concentration (120 mM) was chosen to provide a slight excess over the amount needed to fully oxidize the MoS_2_ nanosheets in one gel according to the presumed reaction stoichiometry: MoS_2_ + 9H_2_O_2_ ⇒ MoO_4_^2−^ + 2SO_4_^2−^ + 6H_2_O + 6H^+^. (B) Cross-linking effects on the full fibrin–MoS_2_ composite gels as a function of genipin loading. The stabilizing effect is dependent on genipin loading and the lifetimes of fibrin–MoS_2_ gels can be extended up to ∼1 week (150 h). Degradation solution contains both enzyme and peroxide under conditions that are the same as panel A, but with solutions changed after each time point.

Genipin is a plant-derived small-molecule known to crosslink diverse biopolymers including chitosan, gelatin, collagen, and fibrin. Genipin is recognized to have much lower toxicity than some other common crosslinking agents like glutaraldehyde, making it an attractive choice for biomedical applications.^78–81^ Here we first incubated the cylindrical gels overnight in genipin/PBS solutions at 37 °C. Fig. 6A shows that genipin cross-linking dramatically increases the resistance of fibrin to degradation. At 1 : 4 genipin : fibrin mass ratio, the gels are resistant to three days of peroxide degradation (80% solids remaining) and enzymatic degradation (72% solids remaining). The formation of new covalent cross-links is likely responsible for the stabilizing effect in both pathways, and since the cross-links are not peptide bonds they are likely unrecognizable to MMPs such as collagenase.

Fig. 6B shows the behavior of the full fibrin/MoS_2_-nanosheet composite gels in the same degradation media and as a function of genipin loading. Here the volumes of the hydrogels were measured *in situ* over time to avoid damaging the gels during extraction as required for mass measurement. Top- and side-view digital photos were taken and used to estimate diameter and height, and an ideal cylinder model used estimate gel volume. The gels can be stabilized for almost 1 week (125 h). Even at the lowest genipin loading (1 : 80 genipin : fibrin mass ratio) the gels persisted for three days, which is a factor of 6 lifetime extension relative to fibrin without genipin cross-linking (Fig. 6). These results demonstrate the ability of genipin not only to extend hydrogel lifetime, but also to preferentially stabilize the fibrin component as desired, and to provide a design tool for tuning the biodegradation time scale through selection of genipin concentration.

Note that genipin addition converts the clear gels to opaque blue/black, which masks any visual evidence for MoS_2_ degradation, such as that seen by the longer term “bleaching” of gel in Fig. 3 (inset). Instead we made ICP-OES measurements of elemental Mo concentration in the degradation solutions (Fig. S7), which shows that MoS_2_ and Mo release occurs gradually during this experiment and is complete at 100–150 h, a time scale similar to complete fibrin degradation in the same experiment. Fig. 6 shows no visual evidence of free nanosheets. Overall, genipin cross-linking is useful both for controlling degradation time scales and for avoiding the nanosheet release (intermediate state B in Fig. 8) as seen previously in unstabilized gels (Fig. 3 lower inset).

#### 2.3.4 Cytotoxicity assays

Fibrin and MoS_2_ were selected for their biocompatibility, but their parallel degradation in these composites produces a complex mixture of soluble molybdates, fibrin degradation products, and possible residues from the genipin cross-linker. Here we assess potential adverse responses to the full degradation product mixtures on primary human ventricular cardiac fibroblasts in culture, an important cell type in cardiac tissue engineering. Cellular viability was assessed with the MTT assay performed on the products of partial degradation of a cross-linked composite gel corresponding to the 52 h time point (blue markers) in Fig. 6. After partial degradation, any remaining macroscopic gel body was removed and the surrounding media concentrated 10× by evaporation, to compensate for a subsequent 10× dilution into the culture media (see Methods). This approach is capable of reproducing the same concentration of degradation products that existed in the original degradation media of 7.3, as a maximum dose that can be further diluted.

**Fig. 7:**
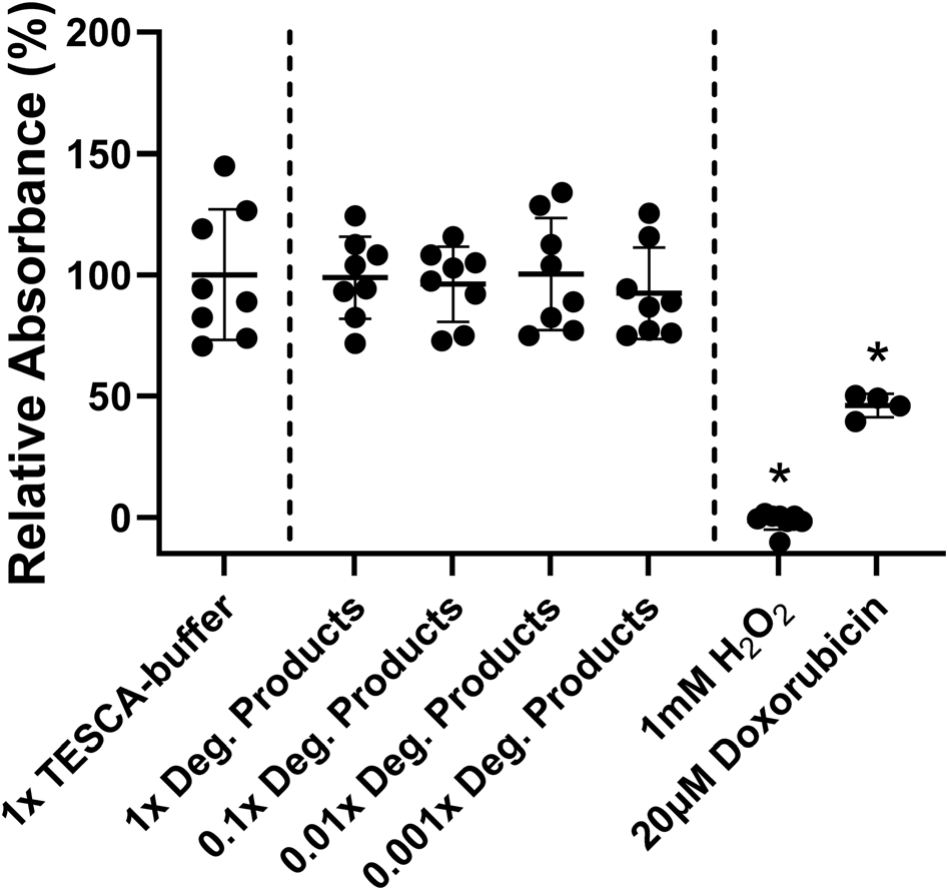
Human cardiac fibroblast viability after 24-hour-long exposure to fibrin/MoS_2_ degradation products at physiologically relevant concentrations. The products were collected from the degradation media after crosslinked gels (0.0125 g genipin per g-fibrinogen) were incubated for two-days with enzyme/peroxide. The specific conditions were the same as those corresponding to the 52 h time point (blue markers) in Fig. 6. Primary human cardiac fibroblasts were cultured for 24 hours with degradation products added to media at 1×, 0.1×, 0.01×, or 0.001× the concentrations in the original degradation medium. Cell viability was measured *via* relative absorbance using a standard MTT assay and compared to TESCA-buffer negative controls as well as hydrogen peroxide and doxorubicin hydrochloride positive controls. Asterisks indicate statistically significant differences (*p* < 0.0001).

Fig. 7 shows the MTT cell viability results for a dose sequence from 0.1% to 100% of the original concentration of products in the degradation media. No statistically significant adverse responses (*p* > 0.9) were seen relative to media control (the TESCA-buffer used in the degradation experiments). Strong adverse responses at (*p* < 0.0001) were seen to 1 mM hydrogen peroxide and 20 μM doxorubicin hydrochloride relative to buffer controls. This result confirms literature expectations of the suitability of fibrin and MoS_2_ as components in biocompatible implants.

## 3. Conclusions

This study introduces a new class of conductive biodegradable hydrogels based on fibrin polymer networks and metallic 1T MoS_2_ as a conductive filler phase. The MoS_2_ nanosheet phase is shown to increase the electrical conductivity by more than a factor of three in the mid-frequency range of 100–1000 Hz. Electrochemical impedance spectroscopy suggests the enhancement is due to the introduction of solid-state conduction routes through a sub-percolation-threshold network of MoS_2_ nanosheets. In pure form, these chemically exfoliated MoS_2_ nanosheets have an electrical conductivity that is 2000× higher, suggesting the potential for greater enhancement through higher filler loadings, if needed in certain applications.

**Fig. 8:**
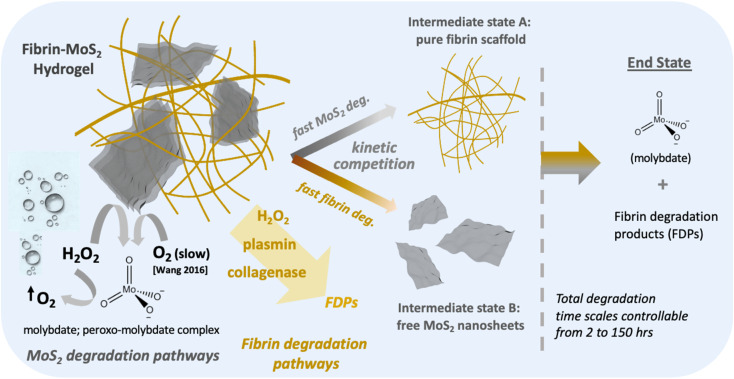
Summary of biodegradation pathways and time scales observed in fibrin/MoS_2_ composite hydrogels.

The biodegradation rates and pathways were studied in detail and shown to be complex for this two-component system (see Fig. 8). Both components (and thus the whole composite) are shown to be biodegradable to soluble products over controllable time scales from 2–150 h. MoS_2_ degrades by O_2_/ROS-mediated oxidation to produce soluble molybdate, which can also catalyze peroxide decomposition and lead to a self-limiting biodegradation reaction when peroxide is the primary oxidant. Fibrin degrades through protease action and can be controlled over a wide range by genipin cross-linking at genipin loadings from 1–25%. Fig. 8 illustrates how the composites can evolve into two very different intermediate states depending on the relative rates of fibrin and MoS_2_ degradation: (A) an intact fibrin network and intact hydrogel monolith after nanosheet degradation of molybdate release (desired for most adhesive or tissue regeneration applications), and (B) a loss of the fibrin network with release of free MoS_2_ nanosheets (of potential interest for some delivery applications). Fibrin cross-linking is demonstrated to be a useful tool controlling the biodegradation pathways to pass through either the A or B intermediate state, as desired. This new class of 2D-enhanced conductive hydrogel are promising for injectable and non-injectable biomedical applications including in the growth, regeneration, or attachment of electrically active tissue.

## 4. Experimental section

### 4.1 Material fabrication and characterization

#### 4.1.1 Nanosheet synthesis

MoS_2_ nanosheets were synthesized by chemical exfoliation of bulk MoS_2_ powder.^31,82^ This procedure yields a mixture of 1T/2H-phase MoS_2_ with the electrically conductive 1T being the majority component.^31^ The chemical intercalant used was *n*-butyllithium, and following reaction with water the nanosheet product was purified by dialysis. The synthesis was carried out in a glove box and the dialysis in an N_2_-purged glove bag to protect MoS_2_ from ambient O_2_ oxidation. The full procedure is given in the SI. The morphology, chemistry, and crystal phase of these nanosheets are reported elsewhere,^31,60^ and the present synthesis batch underwent a quality control check for morphology by high-resolution TEM (JEOL 2100F).

#### 4.1.2 Fabrication of hydrogel monoliths

Cylindrical hydrogel monoliths were fabricated by mechanical mixing of freeze-dried MoS_2_ nanosheets powders with fibrinogen and thrombin solutions in cylindrical molds. Freeze drying of aqueous MoS_2_ stock suspensions (∼1 mg mL^−1^) was used to prevent the sheet restacking that commonly occurs in evaporative drying, and instead creates disordered, low-density, “fluffy” powders that can be more easily resuspended in liquid media. The standard experiment involved suspending 10 mg of freeze dried 1T-MoS_2_ nanosheets in 1 mL of 80 mg per mL fibrinogen in phosphate buffered saline (PBS) in a scintillation vial with sonication. To the vial was then added 1 mL of 0.5 mg per mL thrombin in PBS followed by brief mixing, and gelation was observed to occur within a minute. Some cylindrical hydrogels were post-synthetically crosslinked by genipin by immersing in 17.7 mL of PBS with genipin concentrations of 0.056–1.13 mg mL^−1^ overnight at 37 °C.

#### 4.1.3 Fabrication of hydrogel films

Hydrogel films were fabricated by a dual-barrel syringe method modeled after procedures used in commercial fibrin glues (Fig. 1). Here a 2 mL dual-barreled syringe with a 2 × 8 mm mixing tip (Merlin Packaging) was loaded in one barrel with lyophilized bovine fibrinogen (Sigma) dissolved at 80 mg mL^−1^ in pH 7.4 1 × PBS, and loaded in the other barrel with 40–300 NIH units per mg bovine thrombin powder (Sigma) pre-dissolved in pH 7.4 1 × PBS. Mixing at the syringe tip contacts fibrinogen with thrombin *in situ*, which initiates the polymerization while allowing time for the mixed solution to spread uniformly on polyethylene terephthalate substrates before gelation occurs. In the cases involving MoS_2_ nanosheets, a nanosheet stock solution was pre-mixed with one of two solutions (typically fibrinogen) before loading the syringe.

#### 4.1.4 Hydrogel morphology characterization

The fibrous network structure of the hydrogels was characterized by SEM following glutaraldehyde fixation followed by critical point drying (CPD, Ladd Research Industries) to preserve structure. Hydrogel samples were washed three times in phosphate buffered saline solution (PBS, pH 7.4) and then fixed for 1 h in 4% EM grade glutaraldehyde (EM grade) in PBS. This was followed by dehydration in a series of sequentially more concentrated ethanol solutions: 30%, 50%, 70% followed by three incubations in 100% EM grade ethanol. Dehydrated gels were transferred into anhydrous 100% ethanol in the specimen chamber of a critical-point dryer (CPD, Ladd Research Industries). After sealing the chamber, the ethanol was exchanged by alternately filling the chamber with high pressure liquid CO_2_, waiting 5 minutes for equilibration, then venting and replacing with fresh CO_2_. The dried samples were mounted on aluminum stubs, sputter-coated with gold-palladium (Emitech K550 using Argon vapor lamp), then viewed by scanning electron microscopy (Thermo Apreo VS SEM) operated at 3–5 kV accelerating voltage. The same technique was adopted for films containing MoS_2_ nanosheets except there were increased number of washing steps with PBS prior to glutaraldehyde fixation in order to better rinse out phosphate salt crystals. The successive ethanol concentrations were also carried out with longer equilibration times in order to minimize collapse of the protein around the nanosheets.

#### 4.1.5 Electrical impendence spectroscopy (EIS)

EIS was used to characterize puck-shaped gels in a 2.5 cm diameter cylindrical test cell with top and bottom Ag electrodes in a design similar to Freire *et al.*^4^ or Guarino *et al.*^18^ A hollow polymer cylinder was outfitted with Al foil top and bottom caps whose inner surfaces were coated with conductive silver paste, and the fibrinogen and thrombin suspensions mixed within the cell to form a test cylinder (2–3 mm in height) and gelled *in situ* before capping with the top electrode using the silver paste as both electrode and adhesive layer. The measurement was made using a potentiostat (SP-300, BioLogic) with 0 V bias (*vs.* open circuit voltage) applied along with an AC signal superimposed with 1 mV amplitude in the frequency range of 0.1–200 kHz. The data analysis and equivalent circuit fitting was performed with Z-fit (version 11.43, BioLogic).

### 4.2 *In vitro* biodegradation and toxicity studies

#### 4.2.1 Biodegradation kinetics and intermediates

All hydrogel degradation experiments were carried out on cylindrical hydrogel monoliths synthesized *via* the *ex situ* method. Immediately after synthesis these hydrogels were placed into a 5 mL degradation solution consisting of TESCA buffer containing various degradation reagents. Most common were 40 μL of 30% H_2_O_2_ or 2.5 mg collagenase IA (derived from *Clostridium histolyticum*), with others also used as noted. In 5 mL degradation solutions, this peroxide amount (0.6 mmol) represents a slight excess of the stoichiometric requirement for MoS_2_ oxidation (9 : 1 H_2_O_2_ : MoS_2_ molar ratio), and the collagenase concentration is 0.4 mg mL^−1^. After treatment in this solution for the target time, the structural integrity of the hydrogel, a proxy for degradation of the fibrin scaffold, was assessed by removing the intact portion(s) and transferring them to a laboratory milligram balance for weight measurement in the fully wet state. The monoliths were then not replaced in the degradation medium, but rather separate experiments were performed for each degradation time point. The extent of MoS_2_ degradation was determined by measuring soluble Mo products in the degradation solution by ICP-OES.

#### 4.2.2 Characterization of fibrin degradation products

The molecular weight distribution of the fibrin degradation products was measured by a microfluidic electrophoresis technique. For each of the three primary degradation pathways: enzymatic (collagenase), oxidative (H_2_O_2_) and spontaneous (buffer only), the degradation solutions were analyzed for free protein using a GX Touch II LabChip system (Revvity Inc., Waltham, MA). The standard protocol of the LabChip ProteinExpress assay was used as described elsewhere.^75^ Samples were analyzed in duplicate and representative MW distribution spectra are presented. Specifically, the high sensitivity protocol was followed, which requires 5 μL of sample, using the optional reducing buffer. Each sample was analyzed three times, and each time 20 nL was removed from the well plate onto the detection chip.

#### 4.2.3 Cytotoxicity of degradation products

Primary human ventricular cardiac fibroblasts (Lonza, Cat #: CC-2904, Lot #: 18TL281202) were cultured in DMEM/F12 (Gibco, Cat #: 11320033) supplemented with 10% fetal bovine serum (Life Technologies; Cat #: 16000044), 1% penicillin–streptomycin (Sigma; Cat #: P0781), and 4 ng mL^−1^ basic fibroblast growth factor (Reprocell, Cat #: 030002). Cells were fed every other day until becoming 80 to 90% confluent, at which point they were passaged into 96-well plates using 0.05% trypsin (Gibco, Cat #: 27250-018) in versene (0.5 M EDTA: Cat #: E5134; 1.1 mM d-glucose; Gibco, Cat #: 15023-013) at a density of 5000 cells per cm^2^ (1600 cells per well) for subsequent viability assessment.

The MTT assay was used to test the effects of fibrin/MoS_2_ degradation products on cell viability. Human cardiac fibroblasts were cultured with medias containing soluble degradation products from genipin-crosslinked (1 : 80 genipin : fibrinogen) cylindrical hydrogels that had been exposed to collagenase/H_2_O_2_ solutions (see Fig. 6) for 48 h. TESCA buffer (negative control) or degradation products were concentrated to ten-times their initial concentrations *via* partial evaporation in a 60 °C oven and sterile filtered. Stock solutions were prepared *via* serial dilution of concentrated degradation products with concentrated TESCA buffer to match background. Finally, medias were prepared by diluting the concentrated TESCA buffer or degradation products at 10% v/v in cell culture media such that the highest tested concentration represented the original concentration of degradation products (1×).

Three days after cell plating, the regular culture medium was removed and cells were fed with 100 μL of media containing 1× TESCA-buffer, degradation product (1×, 0.1×, 0.01×, or 0.001×), 1 mM hydrogen peroxide, or 20 μM doxorubicin hydrochloride. After 24 hours, cell viability was measured using a commercially available MTT assay kit (Sigma, Cat #: TOX1-1KT). Briefly, 2-(4,5-dimethylthiazol-2-yl)-2,5-diphenyltetrazolim bromide (MTT) was reconstituted in phosphate buffered saline at a concentration of 5 mg mL^−1^. 10 μL of the MTT solution was added to each well and cells were incubated for an additional 2 hours at 37 °C to allow for formazan crystal formation. After incubation, the MTT-medium mixture was removed from the cells and 100 μL of MTT solubilization solution (Sigma, Cat #: M-8910) was added to each well and gently pipetted up and down and placed on an orbital shaker for 15 minutes to fully lyse the cells and dissolve the formazan crystals. Absorbance was measured at 570 nm and 690 nm (background) using a BioTek™ Cytation 5 Cell Imaging Multimode Reader. Relative absorbance was calculated by subtracting each well's background absorbance from its absorbance at 570 nm followed by subtracting the absorbance difference of media only control wells and dividing by the average of the TESCA-buffer control group.

## Author contributions

Vidushi Shukla: study design, conduct of experiments; writing – early draft; Willis Bilderback: design and conduct of biodegradation experiments and their interpretation; Deisy Fernandes: synthesis and characterization of MoS_2_ and hydrogels; Mark Daley: design and conduct of cytotoxicity experiments; Rojry Basnet: conduct of cytotoxicity experiments; Pushkaraj Joshi: electrical characterization; Zidan Yang, XPS analysis; Anubhav Tripathi: soluble product analysis; Jacob Rosenstein: electrical characterization; Kareen Coulombe: conceptualization, data interpretation; Robert Hurt: conceptualization, data curation, writing – reviewing and editing.

## Conflicts of interest

All the authors declare that they have no conflicts of interest in this work or any commercial or associative interest that represents a conflict of interest in connection with the manuscript.

## Supplementary Material

NA-007-D5NA00377F-s001

## Data Availability

The data for this article are archived in the Brown University Digital Repository and can be accessed at: https://repository.library.brown.edu/studio/item/bdr:tzaqn86t/. SI (additional material characterization, experimental details, alternative graphical representations, and supporting data) is available. See DOI: https://doi.org/10.1039/d5na00377f.
