# Supplementary material for: Fibrin/MoS2-nanosheet conductive hydrogels with programmed time scales and pathways for bioresorption
Source: Nanoscale Adv. 2025 Aug 21;7(19):6205–19. doi: 10.1039/d5na00377f (PMC12379805; doi:10.1039/d5na00377f)
Supplement: NA-007-D5NA00377F-s001 [file NA-007-D5NA00377F-s001.pdf]

Supplementary Information (SI) for Nanoscale Advances.
This journal is © The Royal Society of Chemistry 2025

Supporting Information for

Fibrin/MoS_2-Nanosheet Conductive Hydrogels with Programmed Time Scales and Pathways for Bioresorption

Nanosheet synthesis and material characterization

MoS_2 nanosheets were synthesized by chemical exfoliation of bulk MoS_2 powder as described by Wang et al. [2016]. For that synthesis, 3 ml of 1.6 M n-butyllithium in hexane solution was added to 300 mg MoS_2 powder in an N_2-filled glovebox. The mixture was left under mild stirring at room temperature for 24-48 hours. Still in the glove box, the lithium-intercalated product was rinsed with hexane to remove the excess organo-lithium reagent and organic byproducts. The product was then taken out of the glove box and centrifuged for 30 mins at 4000 rpm. After pouring out the supernatant, the purified intercalated product was exfoliated by reaction with DI water in an ultrasonic bath for 30 minutes. The mixture was then centrifuged at 1000 rpm for 15 mins to get a well-dispersed aqueous dispersion of MoS_2 nanosheets. The supernatant was taken out, leaving behind un-exfoliated MoS_2, and dialyzed in an N_2 filled glove bag against DI water until neutral pH to remove the byproduct LiOH. Samples were stored in an N_2-filled glove box.

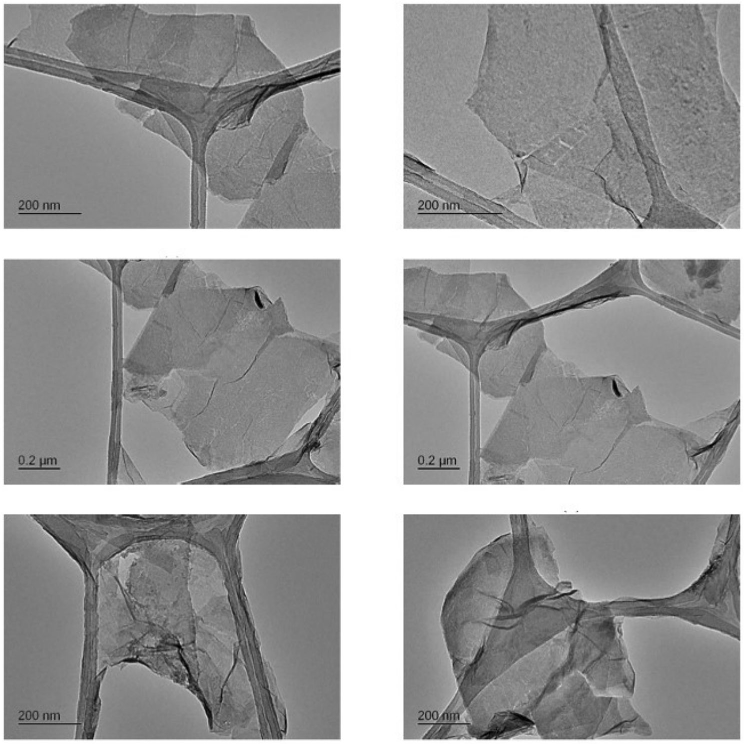

Figure S1. TEM images of as-produced MoS_2 nanosheets on a lacey carbon 300 mesh copper TEM grid. The monolayer nature of the nanosheets is clearly visible, as is some spontaneous wrinkling. A few nanosheets how overlap with other nanosheets, and a smaller number (~10%) show incomplete exfoliation in the form of multilayer nanosheets with multilayer wrinkling.

Figure S2 shows results of XPS characterization of the chemically exfoliated MoS_2 nanosheets. In the S 2p spectrum, the 2H phase is characterized by the $2p_{3/2}$ and $2p_{1/2}$ doublet at ~162.2 eV and ~163.7 eV, respectively, while the 1T phase exhibits corresponding peaks at ~161.3 eV and ~162.3 eV. In the Mo 3d region, the 2H phase is identified by Mo^{4+} $3d_{5/2}$ and $3d_{3/2}$ peaks at ~228.9 eV and ~231.9 eV, respectively, whereas the 1T phase is distinguished by peaks at ~228.4 eV and ~231.5 eV. These binding energy assignments are consistent with previous reports [Guardia et al., 2014; Wang et al., 2016, Eda et al., 2011].

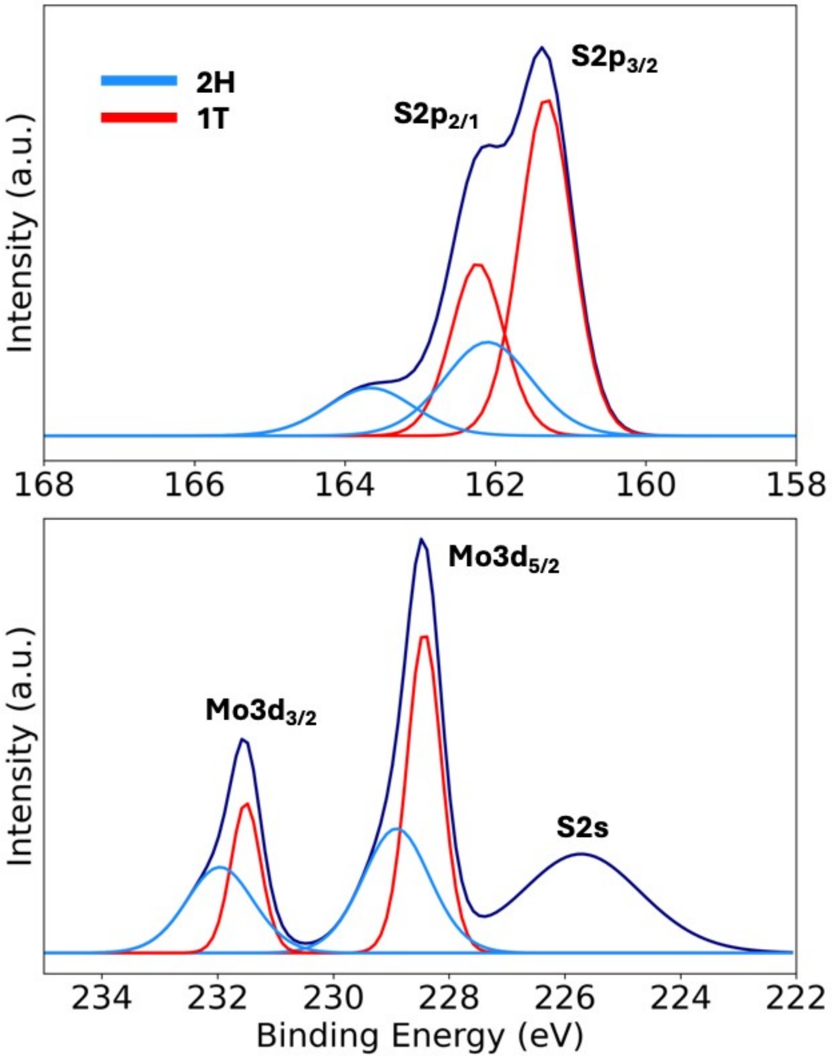

Figure S2. X-ray photoelectron spectroscopy (XPS) results for chemically exfoliated MoS_2 nanosheets. The characterization was performed using a Thermo Fisher Scientific K-Alpha spectrometer. Deconvolution of the S 2p and Mo 3d regions confirms the coexistence of both 1T and 2H polymorphs, with the 1T being the majority phase. More detailed characterization of the products of this synthesis procedure can be found in Wang et al., 2016, 2017.

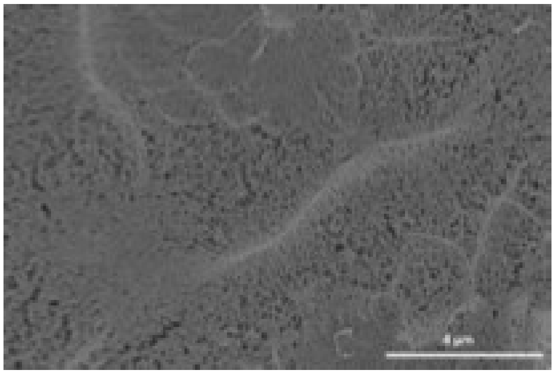

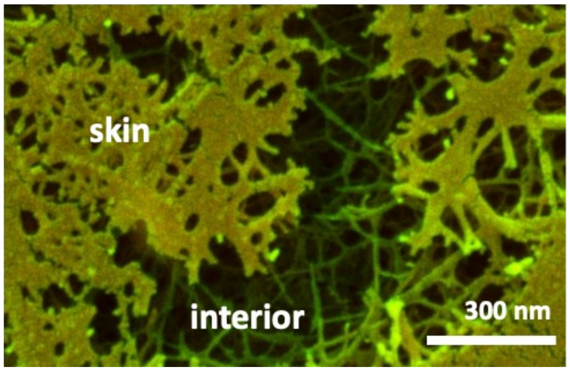

Figure S3. SEM images of a pure fibrin hydrogel thin films. Left: top view, Right: higher magnification showing a skin structure on its top surface consisting of more densely packed fibrin nanofibers aligned in the image plane and a less dense network of random nanofibers in the interior. Sample preparation by critical point drying.

T-peel adhesion screening tests

Fibrin/MoS_2 gel films are of interest as bio-adhesives or sealants where electrical conductivity can enhance tissue growth or integration. In order to evaluate various options for introducing MoS_2 nanosheets into a double barrel mixing syringe protocol, a simple T-peel test was performed to assess the implications on adhesion strength as a marker for the quality of the assembled fibrin network. For this purpose, strips (2 cm x 10 cm) of polyethylene terephthalate (MFLabel) were cut and treated with oxygen plasma (Harrick Plasma PDC-001) to serve as substrates for adhesion testing. Hydrogel films were drop cast on the strips using the dual barreled mixing syringe, and a second PET strip was laid on top of the sample before the hydrogel set. Samples were left to dry overnight in air at room temperature before testing. The joined strips were peeled in an Instron 5940 with a 10 N load cell, and the force-time-profiles averaged and presented as average load (Fig. S4). The measured loads indicate adhesive strengths and can also be used as indicators of the quality of the fibrin network that forms under different synthesis protocols. The fibrin-only reference films yielded average peel loads between 0.28 and 0.37 Newtons (protocol 1). Adding thrombin to the MoS_2 nanosheet suspension (instead of PBS) in one syringe barrel greatly reduces the load (Fig. S4), and visibly degrades shape stability, indicating incomplete gelation (protocol 2). Thrombin is activated by sodium ion, which is abundant in PBS, and auxiliary experiments reveal that reduced Na^+ in the MoS_2 stock relative to PBS is a likely cause of the poor gelation. To restore the Na^+ activity we added sodium salts directly to the MoS_2 stock, but this led to aggregation and flocculation of the nanosheets, which is consistent with a -/- electrostatic stabilization mechanism for MoS_2 nanosheet colloid. To address this problem, we moved the MoS_2 nanosheets to the fibrinogen barrel by adding powdered fibrinogen to the nanosheet stock (protocol 3). This caused some flocculation, but to a lesser extent and the gel set and showed adhesive strengths similar to control. Colloidal stabilization by fibrinogen corona formation has been reported previously in the literature (see main text). We also tried adding sodium bicarbonate as an alternative source since bi-carbonate can act as a deflocculation agent, giving similar results (protocols 4,5). MoS_2 did not enhance adhesion strength in any case, but all cases employing PBS-level Na salts and with MoS_2 separated from the thrombin in the fibrinogen barrel (protocols

3,4, and 5) gave acceptable adhesion strengths similar to the fibrin-only case, which is the formulation used currently in surgical fibrin glue.

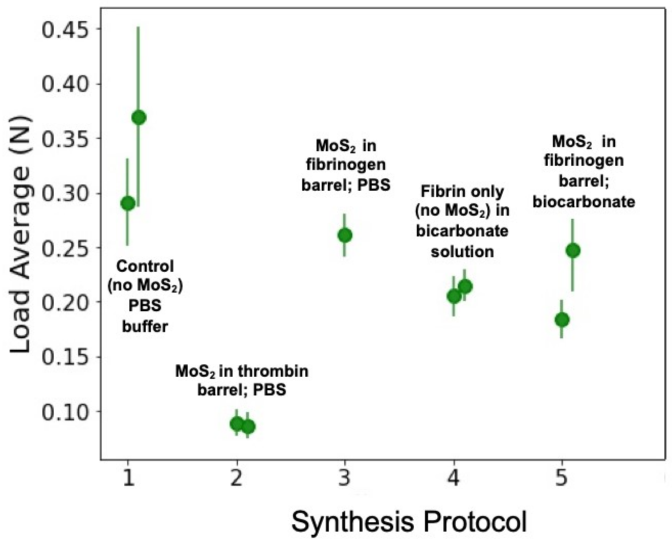

Figure S4. Strength of hydrogel films as adhesives between model polymer substrates as a function of different hydrogel processing methods. All films made by the dual-barreled syringe method, but with different component combinations and buffer solutions in the two barrels.

Electrical characterization

Figure S5 shows the Nyquist plot that corresponds to the Bode plots and circuit analysis shown in the main article (Fig. 2).

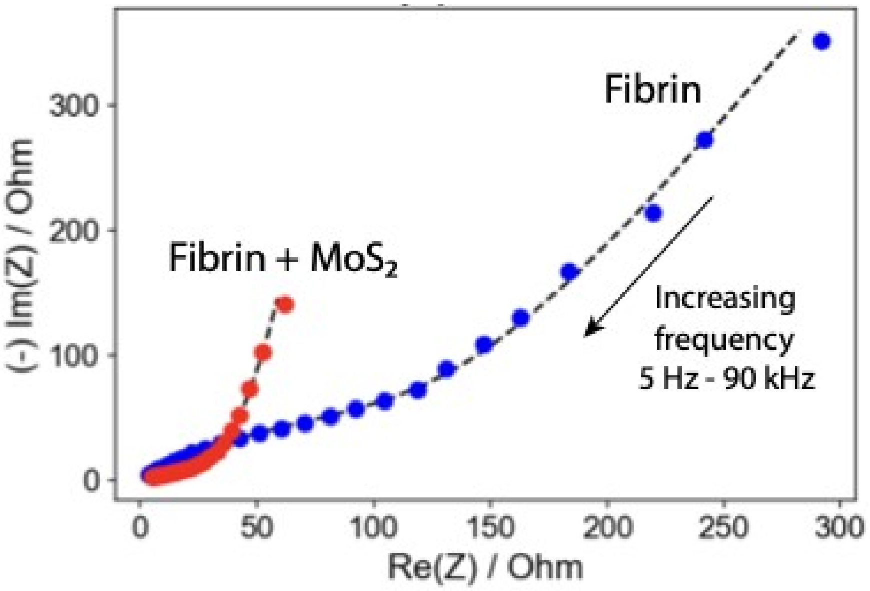

Fig S5: The Nyquist plot demonstrating the electrochemical impedance characteristics of the fibrin hydrogel and the fibrin-MoS_2 composite hydrogel in the frequency range of 5Hz - 90 kHz.

Mechanism of the molybdate - H_2O_2 interaction

Figure S6 shows a proposed catalytic cycle for the observed disproportionation of hydrogen peroxide in the presence of aqueous molybdate. Previous studies confirm that sodium molybdate catalytically disproportionates hydrogen peroxide [Nardello et al. 1995] and also show that diperoxomolybdate complexes exist in the concentration range of H_2O_2 and molybdate used in this system [Aubry and Bouttemy 1997], which forms the basis for the proposed mechanism.

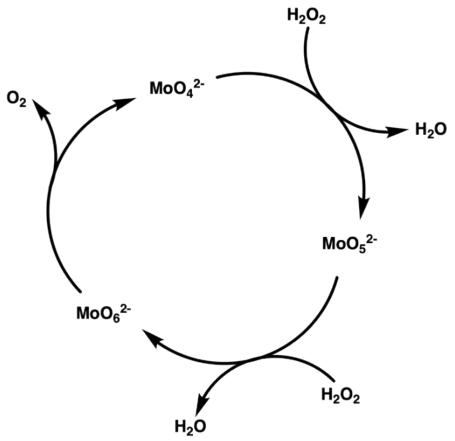

Figure S6.
Proposed catalytic cycle resulting in the disproportionation of H_2O_2 in the presence of MoO_4^{2-}

Molybdenum release during gel degradation

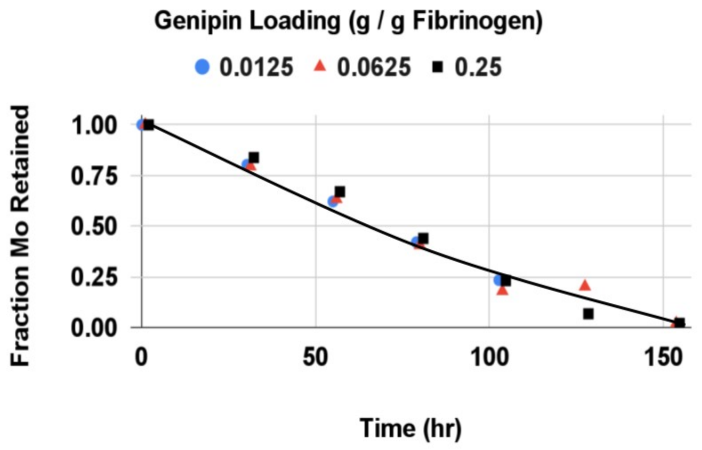

Figure S7. Degradation kinetics for the MoS_2 nanosheet component in composite hydrogels. Time-resolved measurements of soluble Mo release as function of different extents of genipin cross-linking. The Mo release is calculated from ICP measurements of soluble Mo in the degradation fluid, and report as fraction Mo retained in the solid

Supplementary References

Aubry J-M, Bouttemy S, "Preparative Oxidation of Organic Compounds in Microemulsions with Singlet Oxygen Generated Chemically by the Sodium Molybdate/Hydrogen Peroxide System" *J. Am. Chem. Soc.* 119, 23, 5286-5294 (1997).

Eda G, Yamaguchi H, Voiry D, Fujita T, Chen M, Chhowalla M. "Photoluminescence from Chemically Exfoliated MoS_2." *Nano Lett.* 11(12):5111–6 (2011).

Guardia L, Paredes JI, Munuera JM, Villar-Rodil S, Ayán-Varela M, Martínez-Alonso A, et al. Chemically Exfoliated MoS2 Nanosheets as an Efficient Catalyst for Reduction Reactions in the Aqueous Phase. ACS Appl Mater Interfaces. 2014 Dec 10;6(23):21702–10.

Nardello V, Marko J, Vermeersch G, Aubry JM, "^{95}Mo NMR and Kinetic Studies of Peroxomolybdic Intermediates Involved in the Catalytic Disproportionation of Hydrogen Peroxide by Molybdate Ions" *Inorg. Chem.* 34, 20, 4950-4957 (1995).

Wang Z, von dem Bussche A, Qiu Y, Valentin TM, Gion K, Kane AB, Hurt RH, "Chemical dissolution pathways of MoS_2 nanosheets in biological and environmental media", *Enviro Sci & Technology*, 50, 7208–7217 (2016).

Wang, Z.; Zhang, Y.-J.; Liu, M.; Peterson, A.; Hurt, R.H.; "Oxidation suppression during hydrothermal phase reversion allows synthesis of monolayer semiconducting MoS_2 in stable aqueous suspension", *Nanoscale,* 9, 5398 – 5403 (2017).
